# Supplementary material for: Phosphorylation of LKB1 by PDK1 Inhibits Cell Proliferation and Organ Growth by Decreased Activation of AMPK
Source: Cells. 2023 Mar 6;12(5):812. doi: 10.3390/cells12050812 (PMC10000615; doi:10.3390/cells12050812)
Supplement: Supplementary file 1 [file cells-12-00812-s001.zip › cells-1898513-supplementary.pdf]

Supplementary Figure S1. RMSD of the tested LKB1 proteins.

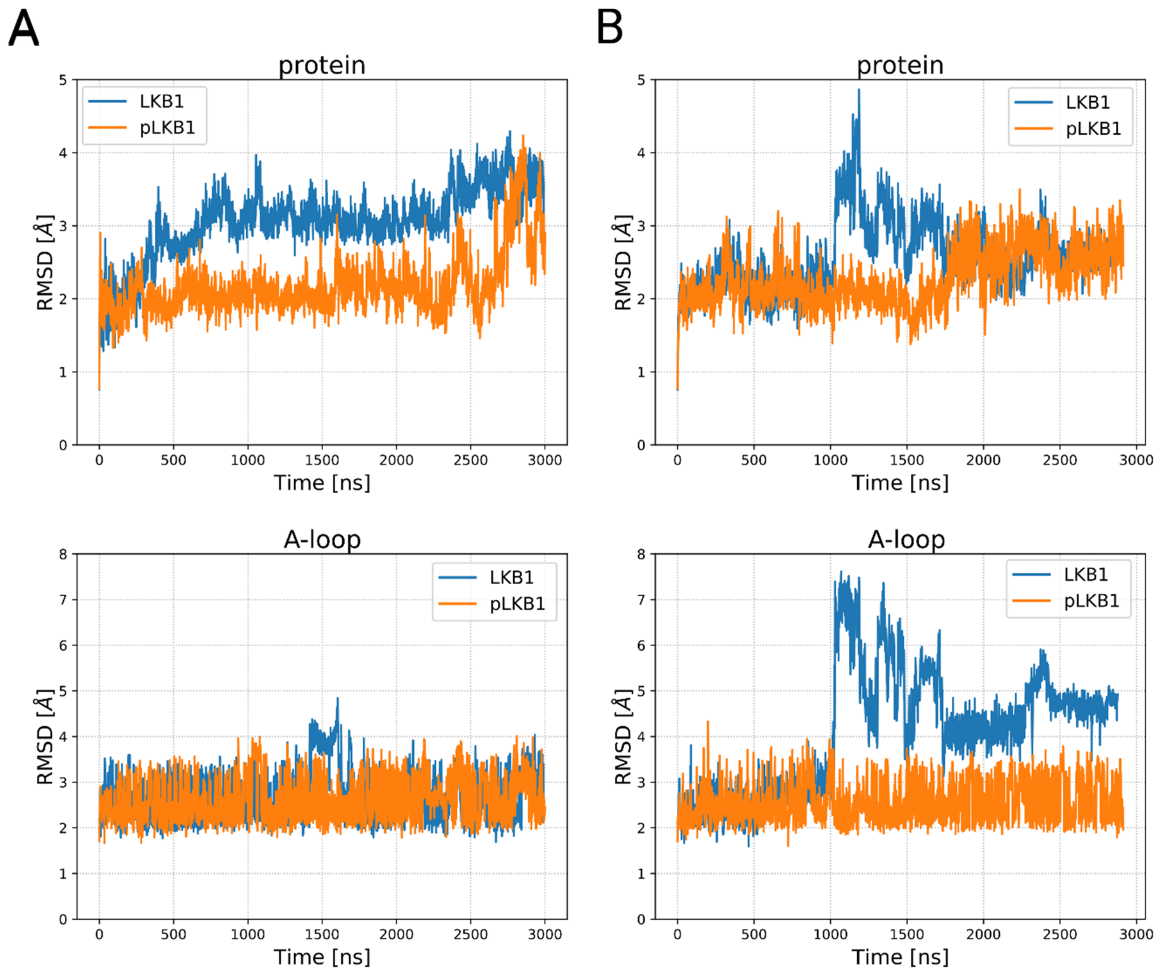

The RMSD of the protein (upper panel) and the A-loop region (lower panel) is shown for the first sample (A) and the second sample (B).

Supplementary Figure S2. Distances between pairs of residues in the binding pocket.

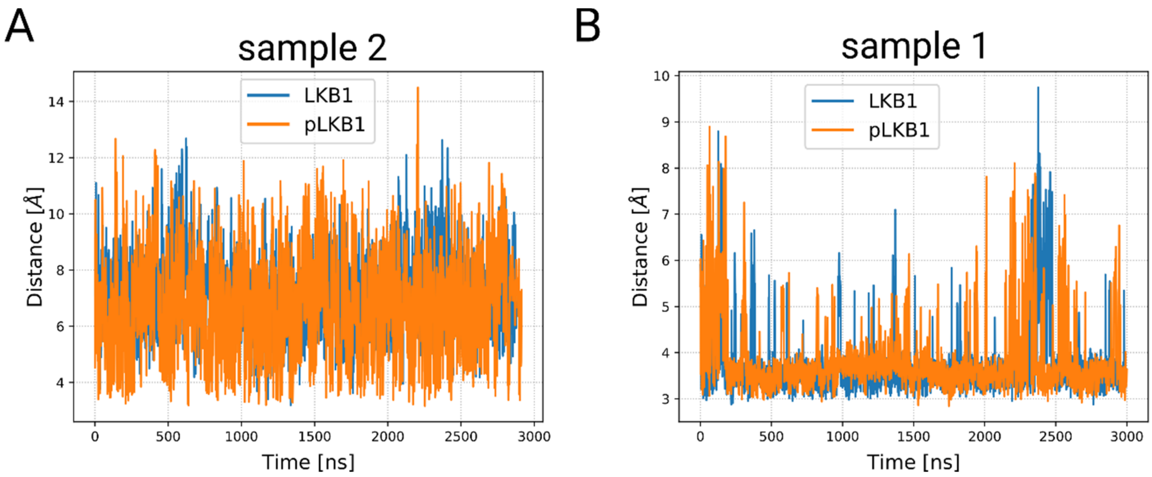

(A) The distance between S60 and A194 residues in the second sample and (B) the distance between K78 and E98 residues in the first sample is represented.
